# Supplementary material for: Evaluation of Toxicity and Efficacy of Inotodiol as an Anti-Inflammatory Agent Using Animal Model
Source: Molecules. 2022 Jul 23;27(15):4704. doi: 10.3390/molecules27154704 (PMC9331631; doi:10.3390/molecules27154704)
Supplement: Supplementary file 1 [file molecules-27-04704-s001.zip › molecules-1796447-SI.pdf]

Supplementary

Evaluation of toxicity and efficacy of inotodiol as an anti-inflammatory agent using animal model

Thi Minh Nguyet Nguyen [1], So-Young Ban [1,2], Kyu-Been Park [1], Chang-Kyu Lee [1], Seoung- Woo Lee[3], Young-Jin Lee[3], Su-Min Baek[3], Jin-Kyu Park[3], My Tuyen Thi Nguyen[4], Jaehan Kim[4], Jihyun Park [1,*] and Jong-Tae Park [1,2,*]

[1] CARBOEXPERT Inc., Daejeon 34134, Korea; nguyet@carboexpert.com, syban@carboexpert.com, kbpark@carboexpert.com, cklee@carboexpert.com

[2] Department of Food Science and Technology, Chungnam National University, Daejeon 34134, Korea

[3] Department of Veterinary Pathology, College of Veterinary Medicine, Kyungpook National University, Daegu 41566, Korea; pyrk2000@gmail.com, bnm3448123@naver.com, suminbaek@naver.com, jinkyu820@knu.ac.kr

[4] Department of food and nutrition, Chungnam National University, Daejeon 34134, Korea; mytuyen108@gmail.com, jaykim@cnu.ac.kr

* Correspondence: Correspondence: jtpark@cnu.ac.kr (J.–T.P.); jane.park7434@gmail.com

Chemical composition of INO20

The chemical composition of INO20 was analyzed by LC-MS/MS in positive mode. Eight compounds were found in INO20 (Fig. S1). The eight compounds have m/z of 393.3, 423.3 (2 compounds), 425.3 (2 compounds), 457.4, 457.2, and 471.2 m/z. Among eight compounds, inotodiol (peak no. 5; 425.3 m/z, RT: 10.9 min) was the major component in INO20. Based on peak area, inotodiol abundance relatively accounted for 71.1 % of the total area. The remaining seven compounds occupied for 28.9% of the total area, and each compound ranged from 1 to 10 %.

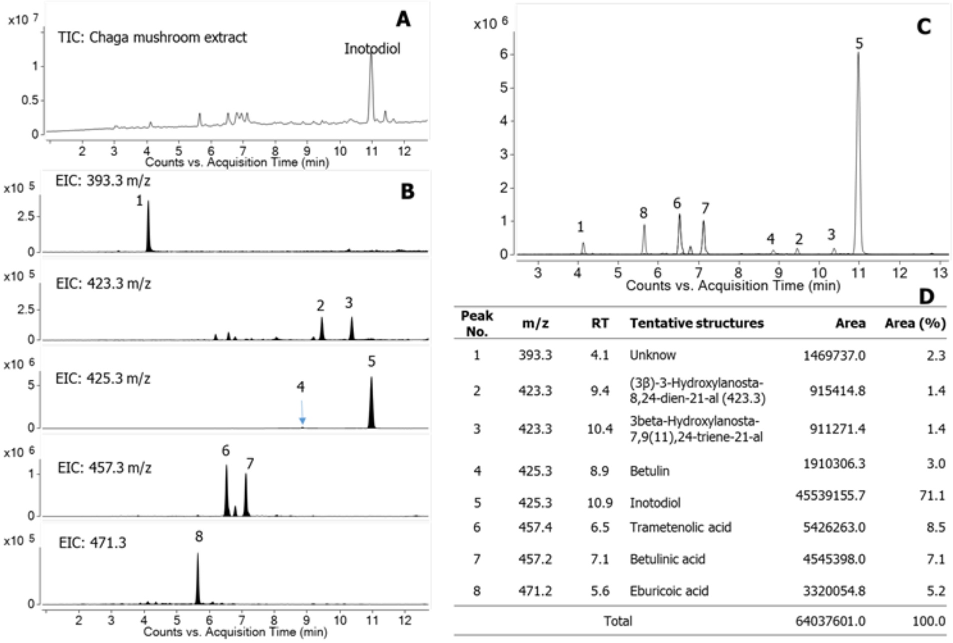

Peak No.	m/z	RT	Tentative structures	Area	Area (%)
1	393.3	4.1	Unknow	1469737.0	2.3
2	423.3	9.4	(3β)-3-Hydroxylanosta-8,24-dien-21-al (423.3)	915414.8	1.4
3	423.3	10.4	3beta-Hydroxylanosta-7,9(11),24-triene-21-al	911271.4	1.4
4	425.3	8.9	Betulin	1910306.3	3.0
5	425.3	10.9	Inotodiol	45539155.7	71.1
6	457.4	6.5	Trametenolic acid	5426263.0	8.5
7	457.2	7.1	Betulinic acid	4545398.0	7.1
8	471.2	5.6	Eburicoic acid	3320054.8	5.2
		Total		64037601.0	100.0

Figure S1. Composition of INO20, analyzed by LC/MS. (**A**) Total ion chromatogram (TIC); (**B**) Extract ion chromatogram (EIC); (**C**) Overlaid EIC; (**D**) Mass-to-charge (m/z), retention time (RT) and area of major compounds.

Cardiotoxicity prediction of inotodiol

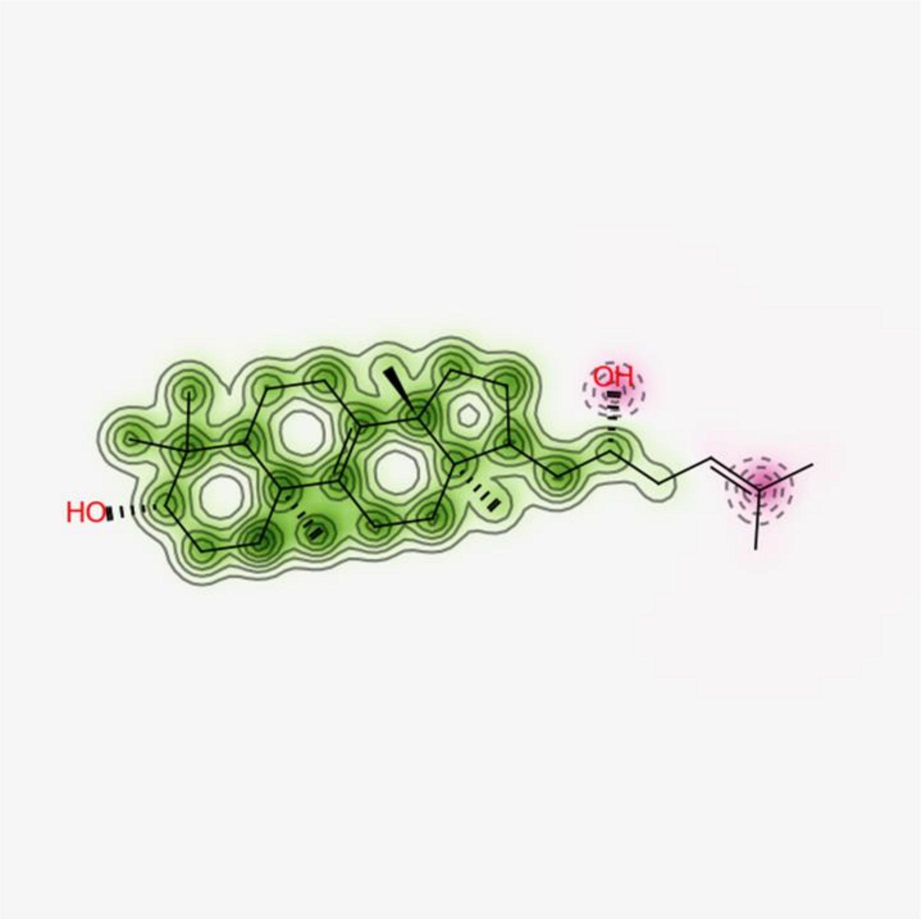

Figure S2. Map of Cardiac toxicity of inotodiol obtained from pred-hERG

Effect of inotodiol on pharmacokinetics

Table S1: Predicted ADME parameters of inotodiol using different prediction webtools

		Inotodiol	
**Properties**	**Parameters**	**pkCSM**	**swissADME**
Absorption	Water solubility	-4.469	-6.25

	Intestinal absorption (human) %	93.178	Low
	Log Kp (skin permeation) cm/s	-2.788	-3.49
	P-gp substrate	Yes	No
Distribution	BBB[a] (log BB)	0.115	No
	CNS permeation (log PS)	-1.796	Not determine
	VD[b] (human) (log L/kg)	0.304	Not determine
Metabolism	CYP1A2 inhibitor	No	No
	CYP2C19 inhibitor	No	No
	CYP2C9 inhibitor	No	No
	CYP2D6 inhibitor	No	No
	CYP3A4 inhibitor	No	No
Excretion	Total clearance (log mL/min/kg)	0.437	0.437
	Renal OCT2 substrate	No	No

[a] Blood-brain barrier, [b] Volume of distribution

pkCSM: Log Kp > -2.5: low skin permeability, Log BB >-0.3: able to cross the BBB, logPS >-2: able to enter the central nervous system (CNS)

Table S2: Predicted human, rat (oral), and environmental toxicity profile of inotodiol

Toxicity	Parameters	Inotodiol
Human	Ames toxicity	No
	hERG I inhibitor	No
	hERG II inhibitor	No

	Hepatotoxicity	Yes
	Max. tolerated dose (human) (log mg/kg/day)	-0.745
Rat (oral)	Oral toxicity (LD50) (mg/kg)	1207
	Oral toxicity classification *	IV
Environmental	*Tetrahymena pyriformis* IGC50-Log10 (mol/L)	0.426
	Fathead Minnow LC50 Log10 (mmol/L)	-0.993

*Class I: Fatal if swallowed (LC50≤5); Class II: Fatal if swallowed (5≤LC50≤50); Class III: toxic if swallowed (50≤LC50≤300); Class IV: harmful if swallowed (300≤LC50≤2000); Class V: may be harmful if swallowed (2000≤LC50≤50000); Class VI: non-toxic if swallowed (LC5≥5000);
